# A CNN-Assisted Enhanced Audio Signal Processing for Speech Emotion Recognition

**DOI:** 10.3390/s20010183

**Published:** 2019-12-28

**Authors:** Mustaqeem, Soonil Kwon

**Affiliations:** Interaction Technology Laboratory, Department of Software, Sejong University, Seoul 05006, Korea; mustaqeemicp@gmail.com

**Keywords:** artificial intelligence, emotion recognition, neural networks, noise removal, spectrogram, signals enhancement

## Abstract

Speech is the most significant mode of communication among human beings and a potential method for human-computer interaction (HCI) by using a microphone sensor. Quantifiable emotion recognition using these sensors from speech signals is an emerging area of research in HCI, which applies to multiple applications such as human-reboot interaction, virtual reality, behavior assessment, healthcare, and emergency call centers to determine the speaker’s emotional state from an individual’s speech. In this paper, we present major contributions for; (i) increasing the accuracy of speech emotion recognition (SER) compared to state of the art and (ii) reducing the computational complexity of the presented SER model. We propose an artificial intelligence-assisted deep stride convolutional neural network (DSCNN) architecture using the plain nets strategy to learn salient and discriminative features from spectrogram of speech signals that are enhanced in prior steps to perform better. Local hidden patterns are learned in convolutional layers with special strides to down-sample the feature maps rather than pooling layer and global discriminative features are learned in fully connected layers. A SoftMax classifier is used for the classification of emotions in speech. The proposed technique is evaluated on Interactive Emotional Dyadic Motion Capture (IEMOCAP) and Ryerson Audio-Visual Database of Emotional Speech and Song (RAVDESS) datasets to improve accuracy by 7.85% and 4.5%, respectively, with the model size reduced by 34.5 MB. It proves the effectiveness and significance of the proposed SER technique and reveals its applicability in real-world applications.

## 1. Introduction

Speech emotion recognition (SER) is the natural and fastest way of exchanging and communication between humans and computers and plays an important role in real-time applications of human-machine interaction. The speech signals generated using sensors for SER is an active area of research in digital signal processing used to recognize the qualitative emotional state of speakers using speech signals, which has more information than spoken words [1]. Many researchers are working in this domain to make a machine intelligent enough that can understand the state from an individual’s speech to analyze or identify the emotional condition of the speaker. In SER, the salient and discriminative features selection and extraction is a challenging task [2]. Recently researchers are trying to finding the robust and salient features for SER using artificial intelligence and deep learning approaches [3] to extracting hidden information, CNN features to trained different CNN models [4,5] to increasing the performance and decreasing the computational complexity of SER for human behavior assessment. In this research era, the SER have faced many challenges and limitation due to the vast users of social media, low coast and fast bandwidth of the Internet. Due to the usage of low-cost internet and social media occur semantic gape. To cover the semantic gap in this area, researchers are worked to covered and introduced new methods to extract the most salient features from speech signals and trained models to accurately recognize the speaker’s emotion during speech. The technology is developed day by day to provide new and flexible platforms for researchers to introduce new methods using artificial intelligence.

The development skills, technology and usage of artificial intelligence and deep learning approaches play a vital role in the enhancement of human-computer interaction (HCI) such as emotions recognition. SER is an effective area of HCI, which has many real-time applications such as it can be used at call centers to identify user satisfaction, human reboot interaction to detect the human emotion, emergency call centers to identify the emotional state of a user for appropriate response and virtual reality. Fiore et al. [6] Implemented SER for car board system to detect the mental condition or emotional state of car drivers to take necessary steps to ensure the safety of passengers. SER is also playing a role in the automatic translation systems and understanding human physical interaction in crowds for violent and destructive actions which difficult to do manually [7]. Badshah et al. [8] used the SER for smart effective services to describe methods using CNN architectures with rectangular shape filters to show the effectiveness of the SER for smart health care centers. Mao et al. [9] improved the effectiveness of SER for real-time applications using the salient and discriminative features analysis, for feature extraction to increase the significance of the HCI. Min et al. [10] used the SER for describing emotion in movies using the content analysis of arousal and violence discriminative features for estimating emotion intensity and emotion type in hierarchically. Miguel et al. [11] used SER for privacy purposes to use the paralinguistic features and privacy-preserving-based hashing method to recognize the speaker.

SER is an emerging area of research where many researchers presented a variety of techniques in this domain. Most researchers are working to find effective, salient, and discriminative features of speech signals for classification to detect the accurate emotion of a speaker. Recently, researchers have used deep learning approaches to detect the salient and discriminative features for SER. High-level features are erected on the topmost of low-level features to perceive and recognize lines, dots, curves, and shapes using convolutional neural networks. The deep learning models (CNN, CNN-LSTM, DNN, DBN, and others) approach to detect the high-level salient features to achieve better accuracy compared to low-level handcrafted features. The usage of deep neural networks boosts the computational complexity of the whole model. There are many challenges in SER domain; (i) current CNN architectures have not revealed any significant improvement in terms of accuracy and cost complexity in speech signal processing. (ii) The usage of RNN and long short-term memory (LSTM) is useful to train sequential data but are difficult to train effectively and are more computational complex. (iii) Most researchers have used the frame-level representation and concatenation methods for feature fusion, which is not suitable for utterance-level SER. (iv) Data sparseness results in large concatenation feature fusion and cannot detect the exact boundary of the word.

Due to the above-mentioned issues and challenges we proposed a novel CNN architecture with special strides rather than a pooling scheme to extract the salient high-level features from spectrograms of speech signals. We detect the hidden patterns of speech signals in convolutional layers that use the special strides for down-sampling the feature maps. The extensive experiments were conducted on two standards benchmarked Interactive Emotional Dyadic Motion Capture (IEMOCAP) [12] and Ryerson Audio-Visual Database of Emotional Speech and Song (RAVDESS) [13] datasets to reveal the significance and efficiency of the suggested model with other states of the art approaches. The detailed experiments and discussion of the proposed method which compares with other baseline methods are mentioned in the experimental section of this paper.

Our major contributions in this article are documented below:

- **Pre-Processing:** SER inputs data in refined form which always need fine-grained form of speech signals to ensure an accurate output prediction of emotions. Existing techniques in SER literature lack the focus on preprocessing steps which effectively refines the data and assists in boosting the accuracy of the final classifier. In this paper, we present a preprocessing strategy where we remove the noises through a novel adoptive thresholding technique followed by silent portions removal in aural data. Thus, our preprocessing strategy plays a prominent role in the overall SER system.
- **CNN Model:** We use the strategy of plain convolutional neural network [14] and proposed a new CNN architecture, DSCNN for SER to learn salient and discriminative features in convolutional layers which uses the special strides within convolutional layer for down-sampling the feature maps rather than pooling layers. The DSCNN model is particularly made for the SER problem using spectrograms.
- **Computational Complexity:** We use minimum convolutional layers in our proposed CNN architectures with small respective fields to learn deep, salient and discriminative features from speech spectrograms to increase the accuracy and achieve reduced computational complexity due to the simple structure of the proposed CNN model, as proved from the experiments.

The rest of the paper is divided as follows: a literature review of SER is explained in Section 2, the proposed framework of SER is described in Section 3, the extensive experimental results and discussion of the proposed technique are mentioned in Section 4, and in Section 5 is the conclusion and an examination of future work of speech emotion recognition.

## 2. Related Work

Digital signal processing is a wide area of research. Recently, researchers have established some efficient techniques in this era for SER using digital audio speech signals to identify the emotional condition of an individual speaker. Robust feature selection, which correctly recognizes the emotions of a speaker, is a challenging task [15] in this domain. A typical SER is divided into two parts: (i) The feature selection process to extract high-level features from speech data; and (ii) the selection of classifiers to correctly recognize emotions from speech. Recently, many researchers have described methods for SER using deep learning approaches to improve the recognition accuracy using audio speech signals, whereas some researchers have used the low-level hand-crafted feature to train CNN, DNN, and NN models to increase the accuracy of SER.

### 2.1. Hand-Crafted Feature-Based Speech Emotion Recognition (SER)

The robust feature selection for SER is a challenging task for researchers. There are some researchers that used the handcrafted features used for SER. Hence, Dave et al. [16] evaluated different features for speech emotions and showed the efficiency for preferable Mel frequency cepstral coefficient (MFCC) [17] features for SER rather than other low-level feature like formant, loudness, linear productivity code (LPC) [18], etc. Liu et al. [19] estimated the gammatone frequency cepstral coefficient (GFCC) features for SER to increase the unweighted accuracy up to 3.6% than MFCCs using additional voice features like jitter and shimmer. Liu et al. [20] proposed a method for SER using a Chinese speech dataset [21] (CASIA), to select hidden emotional features based on correlation and using an extreme learning machine (ELM)-based decision tree for classification. Fahad et al. [22] described a method to select features based on glottal and MFCCs to trained DNN-based models for SER. Wei and Zhao [22] proposed a technique for SER using the autoencoder and sparse classifier to obtain good results on a Chinese speech emotion dataset. The autoencoder extracts the hidden features with large dimensions and sparse network used for extracting sparse features with small dimension to train support vector machine (SVM) classifier to classify the emotions in speech signals.

### 2.2. Convolutional Neural Network (CNN)-Based SER

Recently, researchers have worked with CNN features for SER. Zhang et al. [23] developed a DCNN-based method for SER using the pre-trained CNN AlexNet model to learn deep features and trained a traditional classifier, a SVM, to recognize the emotional state of the speaker. George et al. [24] presented a technique for spontaneous SER-based on CNN and LSTM using the REmote COLlaborative and Affective RECOLA natural emotion database. The author used a CNN model to learn the discriminative feature from whole utterance than fed to LSTM for sequence learning to find the emotions of the speaker. Wen et al. [25] used the random deep belief network for SER. In this method, first they extract the low-level features from speech signals using LLD and fed them to deep belief networks (DBNs) for extracting high-level discriminative features. These high-level features are fed to the SVM classifier, which is connected to each DBN to predict the speaker emotion and then makes decisions based on majority voting. Lian et al. [26] utilized a complicated model, DBN used for features learning to get hidden features from speech and SVM classifier was utilized for emotion prediction to achieve high-level accuracy in SER using CASIA Chinese dataset. Hajar and Hasan [27] proposed a method for SER, to split the speech signals into frames and extracted the MFCCs feature as well as converted them into spectrograms for selecting the keyframe as a whole audio, which represent the utterance of speech. K-mean clustering algorithm is used to select key spectrograms and then train the 3D CNN model to predict the speech emotions. Fei and Liu [28] described a technique for SER using advance long short-term memory (A-LSTM) to learn the sequences using pooling recurrent neural network (RNN) scheme, which gives better performance than simple LSTM. Saurabh et al. [29] used the autoencoder method for speech recognition using the IEMOCAP dataset and evaluated the performance of autoencoder with state-of-the-art GAN [30] in SER.

In the literature, many methods used the CNN model for SER using different types of input, to extract discriminative features from speech signals [9]. In [26,27,28,29,30,31,32] utilized the deep learning approaches for SER to improve the recognition ratio for real-time spontaneous SER using different speech datasets, IEMOCAP, SAVEE, RAVDESS, CAISE TITMIT, etc. To increase the accuracy but the cost computations of the model is also increased due to usage of large pre-trained CNN architectures. Some researchers have developed techniques to recognize speech emotions using spectrograms as an input. Extract features using CNN models and separated classifiers used for classification which boosts the computational complexity of the whole model [31]. In this paper, we introduced a novel CNN architecture for SER using a plain network, which used strides for down-sampling of input features maps in convolutional layers instead of the pooling layer. It reduces the computational complexity of the overall CNN model and improves the accuracy of SER using public benchmark IEMOCAP and RAVDES datasets. The detailed description of the proposed methodology is explained in the subsequent section and efficiency and evaluation of the proposed system are mentioned in the experimental section.

## 3. Proposed Methodology

In this section, we present a CNN-based framework for SER. The proposed framework utilizes a discriminative CNN for feature learning scheme using spectrograms to specify the controversial state of the speaker. The proposed stride CNN architecture has input layers, convolutional layers, and fully connected layers followed by a SoftMax classifier. A spectrogram of the speech signal is a 2D representation of the frequencies with respect to time, that have more information than text transcription words for recognizing the emotions of a speaker. Spectrograms hold rich information and such information cannot be extracted and applied when we transform the audio speech signal to text or phonemes. Due to this capability, spectrogram improve the speech emotion recognition. The main idea is to learn high-level discriminative features from speech signals, for this purpose we utilized a CNN architecture to learn high-level features, the spectrogram is well suited for this task. In [8], the spectrogram and MFCC features are used together using a CNN for SER and classification. In [32] the spectrogram features are used to achieve good performance in SER. The key portion of the recommended framework is described in the following sections.

### 3.1. Pre-Processing

**Figure 1:**
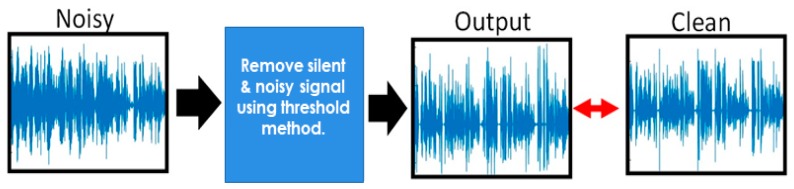
A block diagram of pre-processing to enhance speech signals with an adaptive threshold value.

Pre-processing is an important part of preparing data to achieve model accuracy and efficiency. In this phase, we clean the audio signals to remove the background noises, silent portion and other irrelevant information from speech signal using the adaptive threshold-based preprocessing method [33]. In this method, we find the relationship of energy with amplitude in speech signal using direct relation policy. The energy amplitude relationship is that the amount of energy passed by a wave is correlated to the amplitude of the wave. A high energy wave is considered by a high amplitude; a low energy wave is considered by a low amplitude. The amplitude of a wave mentions the extreme amount of displacement of an element in the middle from its rest location. The logic underlying the energy-amplitude relationship is as follows to remove the silent and unnecessary particle from speech signals. Three steps are included in this process; first, read the audio file step by step with 16,000 sampling rates. In the next step, we find the energy-amplitude relationship in waves and then compute the maximum amplitude in each frame using Equation (1) and passed from a suitable threshold to remove the noises and salient portion and save it in an array. In the last step, we reconstruct a new audio file with the same sample rates without any noise and silent signals. In Equation (1), Ɗ represent the displacements of the particle, f denoted the frequency with respect to time t, and A is a peak of signal or amplitude. The block diagram of the pre-processing is shown in Figure 1.

Ɗ = Ą × sin (2 × π × f × t).(1)

### 3.2. Spectrogram Generation

Dimension of speech signal is one of the challenges tasks in SER using 2D CNN. Since the main aim of this research to learn high-level features from speech signals using the CNN model, so we must convert the one-dimensional representation of the speech signal into an appropriate 2D representation for 2D CNN. Spectrogram is the best and suitable representation of audio speech signals in two dimensions, which represent the strength of speech signals over different frequencies [8].

**Figure 2:**
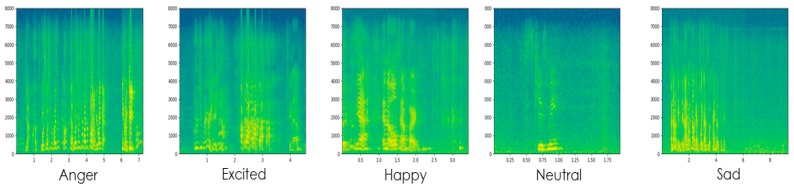
Visual representations of speech signal in 2D spectrograms of various emotions.

The short-term Fourier transformation (STFT) is applied to speech signal for visual representation of frequencies over different times. Applying STFT, to convert longer time speech signal to shorter segment or frame which has an equal length and then applied fast Fourier transformation FFT on frame to compute the Fourier spectrum of that frame. In spectrograms, the time *t* is represented by x-axis and the y-axis represents the frequencies f, of every short time. Spectrogram *S* contains multiple type frequencies f, over different time t, in corresponding speech signal S (t, f). Dark colors in spectrograms illustrate the frequency in a low magnitude, whereas light colors show the frequency in higher magnitudes. Spectrograms are perfectly suitable for a variety of speech analysis including SER [34]. Sample of extracted spectrograms of each audio file by applying STFT are shown in Figure 2.

### 3.3. CNN

CNNs are current state-of-the-art models that are used to extract high-level features from low-level raw pixel information. CNN uses the numbers of kernels to extract high-level features from images and such features is used for training a CNN model to perform significant classification task [34]. CNN architecture is a combination of three components; convolutional layers, which contain some numbers of filters to apply on input. Every filter scans the input using the dot product and submission method to produce the numbers of features maps in a single convolutional layer. The second component is pooling layers, which is used for reducing or down-sampling the dimensionality of features maps. There are some schemes used for reducing dimensionality like; max pooling, min pooling, mean pooling, average pooling, etc. The last component is fully connected layers (FC) of CNN, which mainly used for extracting the global features that are fed to a SoftMax classifier to find out the probability for each class. A CNN arranges these all layers in hierarchical structure, convolutional layers (CL), pooling layers (PL), and then FC followed by the SoftMax classifier. The proposed architectures are explained in the coming section.

### 3.4. Proposed Deep Stride CNN Architecture (DSCNN)

**Figure 3:**
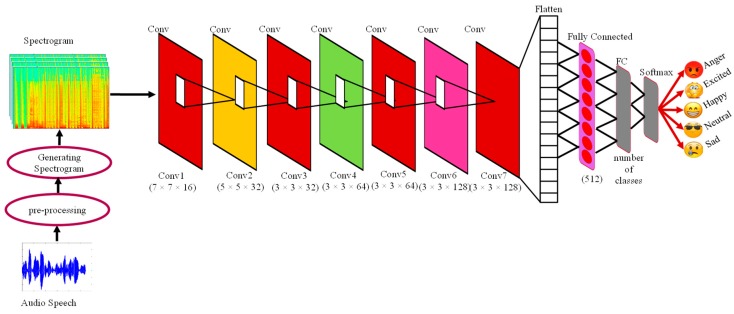
Overall architecture of the proposed deep stride convolutional neural network for speech emotion recognition.

The proposed deep stride CNN model for SER is shown in Figure 3. Our deep stride CNN model is mostly encouraged by the idea of plain nets [14] which are specially designed for computer vision problems, like image classification, localization, tracking, and recognition to secure high-level accuracy [35]. We explore the plain network from image classification to speech emotion recognition and classification. We define the stride deep CNN architecture, which has used mostly the same and small filter size, (3 × 3), to learn deep features with a small respective field in convolutional layers. It follows simple rules, the number of kernels is the same which gives the same output features maps and if the size of the feature maps is reduced to half, the number of filters must be doubled to maintain the time complexity per layer. To follow this strategy, we designed the DSCNN model for SER, which has used the stride (2 × 2) scheme to down-sample the size of features maps directly in convolutional layers rather than the pooling layer. The total number of layers in DSCNN is nine (9), seven (7) convolutional layers, and two (2) fully connected layers are fed to SoftMax for producing the probabilities of speech emotions. The generated spectrograms take as input and applied convolutional filters to extract features maps from a given speech spectrogram.

In the proposed architecture, we consecutively arranged CL. In the first convolutional layer (C1) has 16 number of kernels with squire shape of size (7 × 7) are applied to the input spectrogram with the same padding and stride setting of (2 × 2) pixel. Similarly, in the second convolutional layer (C2) it has 32 filters of size (5 × 5) with (2 × 2) stride setting. The C3 layer uses the same number of filters, stride, and padding as C2, but the size of the filters is 3 × 3. C4 and C5 layers have 64 (3 × 3) kernels with a stride setting of 2 × 2 pixels. In the same way, C6 and C7 layers have 128 kernels of size (3 × 3) with the same stride and padding. The last convolutional layer, which is followed by a flattening layer to convert the data shape into vector form and then the features are fed to FC. The first FC layer has 512 neurons and the last FC layer has the same number of neurons as classes. In the proposed DSCNN uses the rectified linear unit activation function which is followed by batch normalization to regularize the model after every convolutional layer. The first FC is followed by a 25% dropout ratio to deal with the model overfitting [35]. The last FC layer fed to the SoftMax classifier to calculate the probability of each class. The DSCNN is designed for SER using spectrograms. The DSCNN contains convolutional layers and FC layers while eliminating the pooling layer from the whole network and used the same and small filter size and special strides. In first CL has a large kernel size to learn local feature and step-by-step increase the number of kernels but remains same the size and shape of filters to extract the discriminative features from spectrograms. The key component of this architecture is the usage of strides, for down-sampling while eliminating the pooling layer, using the same filter size and shape throughout the network to learn deep features, and limiting the number of FC layers that are used to avoid the redundancy. Due to the above-mentioned characteristics, the proposed DSCNN model captures the robust salient features from spectrograms.

### 3.5. Model Organization and Computational Setup

The recommended DSCNN model layout is implemented in python using the scikit-learn package for machine learning and other resources. The spectrograms are generated from each file, 128 × 128 in size. The whole generated spectrograms are divided by an 80%/20% split ratio for training and testing, respectively. The model training process was evaluated on a single NVIDIA GeForce GTX 1070 GPU with 12 GB of on-board memory for the proposed DSCNN model for SER. The model was trained on 50 epochs with a 0.001 learning rate and a decay one later every 10 epochs. The batch size is 128 in the whole training process and the best accuracy was achieved after 49 epochs with 0.3215 lost on training and 0.5462 lost on validation. The model trains in very little time with a reduced model size (34.5 MB), indicating the computational simplicity.

## 4. Experiments and Results

In this section, we evaluated our SER model on IEMOCAP and RAVDESS datasets using spectrograms. The performance of the proposed CNN models compares with recent CNNs architectures for SER using spectrograms. Several experiments were conducted for SER, the details results are discussed in the subsequent section.

### 4.1. Datasets

#### 4.1.1. Interactive Emotional Dyadic Motion Capture (IEMOCAP)

IEMOCAP is an acted English speech emotion dataset [12] which contains 10 actors to record the different emotions like anger, fear, happy, disgust, sad, neutral, and excited. The IEMOCAP dataset contains 12 h of audiovisual data, which is divided into five sessions, each session has two actors to record a script in multiple emotions. In our work, we used only four emotions anger, happy, neutral, and sad for experimental evaluations to compare with state-of-the-art techniques.

#### 4.1.2. Ryerson Audio-Visual Database of Emotional Speech and Song (RAVDESS)

The Ryerson audiovisual database of emotional speech and song [13] is an English language emotion dataset, which widely used for emotional song and speech recognition. The dataset consists of 24 actors (12 male and 12 female) to record eight (8) different emotions like anger, calm, happy, sad, surprise, disgust, neutral, and fearful. The total number of utterances is 1440 wav files with a 48,000 Hz sampling rate.

### 4.2. Experimental Evaluations

In this section, we evaluated our proposed technique on two benchmark datasets IEMOCAP and RAVDESS. We performed the experiments on two types of spectrograms, raw spectrogram, the audio signals directly converted to spectrograms. Secondly, on the clean spectrogram, the audio signals are processed to remove the silent and noises portion and then converted into spectrograms. We performed utterance-based experiments on SER with a five-fold cross-validation technique. The data was split by 80/20, the 80% data were used for training and 20% for testing the model. We performed two sets of experiments, in the first set, we trained a DSCNN model on raw spectrograms and test the prediction performance for model efficiency and accuracy. In the second set, we trained the model on clean spectrograms and evaluated the performance of the model. Measure the evaluation of the model in term of precision, recall, f1 score, weighted (the correctly predicted emotion samples in a class divided by the total numbers of motion samples in corresponding class) and unweighted (total correct predictions of samples divided by the total numbers of samples in dataset) accuracy. The training results of the proposed model are listed in Table 1, Table 2 and Table 3 and the detail discussion and comparisons are discussed in discussion sections.

Table 1 clearly indicated the difference between raw, and clean spectrograms, on both datasets. That is why the proposed DSCNN model which used strides in convolutional layers [14] to down-sample the features maps rather than pooling layers. The performance is better than the pooling scheme for speech data to recognize emotions using spectrograms.

#### 4.2.1. Results and Performance of DSCNN Model

We used the plain nets [14] CNN architecture to develop a DSCNN model for SER and performs experiments on utterance-based speech spectrograms which is generated from speech signals. The DSCNN model was trained on these generated spectrograms using an 80%/20% splitting approach. The model was trained on two standard benchmark IEMOCAP and RAVDESS datasets and test the prediction performance respectively. Table 2 shows the performance; class level precision, recall, f1 score, weighted and unweighted accuracy of model training on raw and clean spectrograms using the IEMOCAP dataset. Table 3 represents the overall performance, including class level accuracy, precision, recall, f1 score, weighted and unweighted accuracy on raw and clean, spectrograms using the RAVDESS dataset, respectively.

#### 4.2.2. Prediction Performance of Proposed DSCNN

The prediction performance of the proposed DSCNN model is evaluated on IEMOCAP and RAVCDESS datasets to show the efficacy of the system. Table 3 shows the prediction performance of the model in terms of the confusion matrix on the IEMOCAP dataset. Table 4 represents the confusion matrix of the model on the RAVDESS dataset. The prediction performance of the proposed CNN model improves the overall prediction accuracy, which is clearly indicated the significance and robustness of the proposed model.

Table 4 shows the overall prediction performance of each file of four emotions In the IEMOCAP dataset. Even so, the anger and neutral obtained good prediction accuracy and happiness and sadness slightly less, but the overall spectacle of the model (81.75%) is good for the IEMOCAP dataset. Similarly, Table 5 represents the prediction performance of the RAVDESS dataset for eight (8) classes. The model achieves better prediction for all classes in RAVDESS but fearful and disgust are somewhat mixed with clam and anger emotion but the overall prediction accuracy (79.5%) is good for the RAVDESS dataset. The training and testing accuracy of the proposed DSCNN model for both datasets are mentioned in Table 6.

#### 4.2.3. Cross Dataset Experiment

Now a day there are many researchers developed a sufficient technique for SER using single corpus, which obtained a high accuracy in experiments for recognition. When the model is utilizing in natural environment for SER there are many challenges effects the model performance like different type of languages, variety of cultures, huge altering of speakers. We evaluate our model effectiveness using cross dataset performance [36] between IEMOCAP and RAVDESS dataset for four emotions. We train the model on IEMOCAP dataset and test the trained model on RAVDESS dataset. The confusion matrix of cross datasets is presented in Table 7.

Table 7 presents the performance of the proposed model over cross dataset that indicate the effectiveness of the model to recognize the four emotions with 56.5% average recognition rate. The anger emotion gets 77%, neutral 56%, sad 49%, and happy 44% rate of emotion recognition in cross corpus respectively. The cross-dataset experiments show the robustness and significance of the model.

### 4.3. Discussion

The architecture of the DSCNN model and adoptive threshold-based preprocessing of the speech signal to remove the noise and unimportant portion from utterances are major contributions in this work. Our deep stride CNN (model) is mostly encouraged by the idea of plain nets [14] which are specially designed for computer vision problems, like image classification, localization, tracking, and recognition to achieves high-level accuracy [37]. We explore the plain network for SER and define the stride deep CNN architecture. It has used mostly the same and small filter size (3 × 3) with increasing the number of kernels in CL, respectively, and follow simple rules. The number of kernels is the same which gives the same output features maps. If the size of the features maps is reduced to half, the number of filters must be doubled to maintain the time complexity per layer. We have used the stride (2 × 2) to down-sample the size of features maps directly in convolutional layers rather than the pooling layer. The total number of layers in DSCNN is nine (9), seven (7) CL, and two (2) FC layers are fed to SoftMax layer for prediction of the probabilities of speech emotions. The worth of our model is that it has fewer convolutional layers, same and small kernels size, to learn deep salient features with small respective field. Lower complexity than other state-of-the-art deep-learning approaches and achieves high accuracy for SER. The pooling scheme gives good results in computer vision problems, like image recognition, tracking, and retrieval but it is not more robust in signal processing. Thus, the stride CNN model results outperformed other state-of-the-art methods.

Table 8 represents the performance of the proposed method compared with other state-of-the-art, which outperforms the existing results over IEMOCAP dataset using utterance level speech spectrograms. Haytham et al. [38] described a neural network and RNN-based technique for SER using spectrograms of frames to train DNN, which has a high computational coast and did not achieve high accuracy. Tripathi et al. [43] described the deep learning models for SER using transcript and phoneme. To train the different models with different features to increase the accuracy up to 71%, but they used same architecture, which is used for computer vision-related tasks. Chen et al. [42] developed a system for SER using 3D CNN architecture and trained model to increase the accuracy of SER, but he also used the pooling scheme to develop the network. Due to this limitation, we explored the plain CNN architecture to propose a new model for SER to give well and outperform results from state-of–the-art.

Table 9 shows the comparison of the proposed framework with the baseline method on the RAVDESS speech emotion dataset. It represents the significance and efficiency of the proposed DSCNN model on the RAVDESS dataset, which outperformed results in SER. Yuni et al. [43] presented a spectrogram-based CNN model for multi-class audio classification on the combination of two models to achieve 64.48%, accuracy in multitask SER. Jalal et al. [44] and Anjali et al. [45] used the log spectrogram and spectral feature to recognize the emotion in speech data with 68% and 75%, accuracy, respectively. Table 10 shows the computational simplicity of the proposed DSCNN model with others baseline CNN model using IEMOCAP dataset for SER.

The proposed DSCNN model also compared with state-of-the-art CNNs model with respect to training time, model size and accuracy over IEMOCAP and RAVDESS datasets for SER. Table 10 shows the success and significance of the proposed model with baseline methods. It represents the computational simplicity of the proposed model in terms of training time, model size, and accuracy on generated spectrograms of speech signals. We train the AlexNet, Vgg-16 [47], and Resnet-50 [48] CNNs models with transfer learning techniques using the IEMOCAP dataset. The proposed DSCNN model showed outperformed results and proved the effectiveness and significance of the proposed CNN model for SER.

## 5. Conclusions

The literature of SER faces too many challenges to improve recognition accuracy as well as to decrease the computational complexity of the overall model. Due to these challenges, we proposed a CNN architecture with some salient features extraction mechanism to improve the accuracy and achieve reduced computational complexity of the overall SER model. In this paper, we used a dynamic adaptive threshold technique to remove noise and silent signals from speech signals. Then the enhanced speech signals are converted to spectrograms to increase the accuracy and decrease the computational complexity of the proposed model. We used stride CNN architectures for SER using spectrograms to learn most salient and discriminative features in a convolutional layer using some special stride set to down-sample feature maps rather than pooling layers. The effectiveness of the proposed model is evaluated on two standard benchmark IEMOCAP and RAVDESS datasets. The results are convincing and able to recognize emotions in speech signals. Our method achieves accuracy up to 79.5% using RAVDESS and 81.75% on an IEMOCAP dataset with lesser number of parameters in the used model, yielding a computational-friendly output system. This indicates the significance and effectiveness of the proposed system for SER using spectrograms of speech signals.

## Figures and Tables

**Table 1:** Comparison of the proposed model, using raw spectrograms and clean spectrograms.

Model	Input	Dataset	Weighted Acc%	Unweighted Acc%	F1 Score%
Model	Raw spec	IEMOCAP	76	72	77
Model	Clean spec	IEMOCAP	84	82	84
Model	Raw spec	RAVDESS	68	61	70
Model	Clean spec	RAVDESS	80	79	81

**Table 2:** Training performance of the proposed DSCNN model on raw and clean spectrograms using IEMOCAP.

Nature	Result on Raw Spectrograms			Result on Clean Spectrograms		
Emotion	Precision	Recall	F1 Score	Precision	Recall	F1 Score
Anger	0.96	0.87	0.91	0.87	0.96	0.91
Happy	0.58	0.85	0.69	0.97	0.68	0.80
Neutral	1.00	0.76	0.86	0.77	0.91	0.83
Sad	0.69	0.92	0.79	0.82	0.92	0.84
Weighted Avg	0.80	0.77	0.76	0.86	0.85	0.85
Unweighted Avg	0.76	0.73	0.72	0.86	0.86	0.82
**Accuracy**	**-**	**-**	**0.77**	**-**	**-**	**0.84**

**Table 3:** Training performance of the proposed DSCNN model on raw and clean spectrograms using RAVDESS.

Nature	Result on Raw Spectrograms			Result on Clean Spectrograms		
Emotion	Precision	Recall	F1 Score	Precision	Recall	F1 Score
Anger	0.40	1.00	0.57	0.79	0.91	0.84
Happy	0.92	0.29	0.44	0.79	0.90	0.84
Neutral	0.91	0.42	0.57	0.71	1.00	0.83
Sad	0.98	0.98	0.98	0.90	0.96	0.93
Clam	0.82	0.75	0.78	0.71	0.94	0.81
Fearful	0.00	0.00	0.00	1.00	0.50	0.67
Surprised	0.90	0.46	0.61	0.89	0.87	0.88
Disgust	0.92	0.86	0.89	1.00	0.38	0.55
Weighted Avg	0.79	0.70	0.68	0.85	0.81	0.80
Unweighted Avg	0.73	0.59	0.61	0.85	0.81	0.79
**Accuracy**	**-**	**-**	**0.70**	**-**	**-**	**0.81**

**Table 4:** Confusion matrix for emotions prediction on IEMOCAM with average recall value (81.75%) and each row indicated the confusion of each emotion with ground truth and predictions.

Emotion Class	Anger	Happy	Neutral	Sad
Anger	0.90	0.06	0.01	0.03
Happy	0.09	0.74	0.08	0.09
Neutral	0.00	0.07	0.90	0.02
Sad	0.00	0.02	0.25	0.73
**Overall Accuracy**				**81.75%**

**Table 5:** Confusion matrix for emotions prediction on RAVDESS with average recall value (79.5%) and each row indicated the confusion of each emotion with ground truth and predictions.

Emo Class	Anger	Clam	Disgust	Fear	Happy	Neutral	Sad	Surprised
Anger	0.82	0.00	0.00	0.00	0.15	0.00	0.00	0.03
Clam	0.00	0.85	0.00	0.00	0.00	0.15	0.00	0.00
Disgust	0.18	0.16	0.52	0.00	0.00	0.05	0.00	0.09
Fear	0.21	0.14	0.00	0.43	0.07	0.11	0.00	0.04
Happy	0.04	0.00	0.00	0.00	0.87	0.02	0.02	0.04
Neutral	0.00	0.03	0.00	0.00	0.00	0.95	0.00	0.03
Sad	0.00	0.00	0.00	0.00	0.02	0.04	0.94	0.00
Surprised	0.00	0.02	0.00	0.00	0.00	0.00	0.00	0.98
**Overall Accuracy**								**79.5%**

**Table 6:** Training and testing accuracy of the proposed DSCNN model.

Dataset	Training Accuracy	Testing Accuracy
**IEMOCAP**	84%	81.75%
**RAVDESS**	81%	79.50%

**Table 7:** Confusion matrix for emotions prediction on cross corpus between IEMOCAP and RAVDESS.

Emotion Class	Anger	Happy	Neutral	Sad
Anger	0.77	0.08	0.05	0.09
Happy	0.11	0.44	0.01	0.43
Neutral	0.01	0.10	0.56	0.33
Sad	0.00	0.51	0.00	0.49
**Overall Accuracy**				**56.5%**

**Table 8:** Comparison of the proposed method with base line method using IEMOCAP dataset.

Method	Input	Weighted Accuracy	Unweighted Accuracy	Accuracy
Fayek et al. [38]	Spectrograms	64.78%	60.89%	-
Luo et al. [39]	Spectrograms	60.35%	63.98%	-
Tripathi et al. [40]	Spectrograms	71.3%	61.6%	-
Yenigalla et al. [41]	Spectrograms	73.9%	68.5%	-
Chen et al. [42]	Spectrograms	-	-	64.74%
Proposed model	Raw_Spectrograms	**76%**	**72%**	**73.8%**
Proposed model	Clean_Spectrograms	**84%**	**82%**	**81.75%**

**Table 9:** Comparison of the proposed method with base line method using RAVDESS dataset.

Method	Input	Weighted Accuracy	Unweighted Accuracy	Accuracy
Zeng et al. [43]	Spectrograms	-	-	64.48%
Jalal et al. [44]	log-spectrogram	-	69.4%	68.10%
Bhavan et al. [45]	spectral features	-	-	75.69%
Proposed model	Raw_Spectrograms	68%	61%	70.00%
Proposed model	Clean_Spectrograms	80%	79%	79.5%

**Table 10:** Computational comparison of the suggested DSCNN model with other baseline CNNs models.

Model	Training Time	Model Size	Accuracy
Alex Net (transfer Learning) [46]	38 min	201 MB	70.54%
Vgg16 (transfer Learning) [37]	55 min	420 MB	73.00%
ResNet50 (transfer Learning) [14]	30 min	75 MB	75.50%
Proposed DSCNN model	**14 min**	**34.5 MB**	**81.75%**

## Abbreviation

HCI	Human–computer interaction
SER	Speech emotion recognition
MFCC	Mel frequency cepstral coefficient
LLD	Low level descriptors
CNN	Convolutional neural network
DSCNN	Deep stride convolutional neural network
CL	Connected layers
FC	Fully connected layers
SDFA	Salient discriminative features analysis
STFT	Short-term Fourier transformation
FFT	Fast Fourier transformation
IEMOCAP	Interactive emotional dyadic motion capture
RAVDESS	Ryerson audio visual database of emotional speech and song
